# Are Prostate Specific-Antigen (PSA) and age associated with the risk of ISUP Grade 1 prostate cancer? Results from 72 996 individual biopsy cores in 6 083 men from the Stockholm3 study

**DOI:** 10.1371/journal.pone.0218280

**Published:** 2019-06-13

**Authors:** Thorgerdur Palsdottir, Tobias Nordström, Markus Aly, Johan Lindberg, Mark Clements, Lars Egevad, Henrik Grönberg, Martin Eklund

**Affiliations:** 1 Department of Medical Epidemiology and Biostatistics, Karolinska Institutet, Stockholm, Sweden; 2 Department of Clinical Sciences, Danderyd Hospital, Stockholm, Sweden; 3 Department of Urology, Karolinska University Hospital Solna, Stockholm, Sweden; 4 Department of Oncology-Pathology, Karolinska Institutet, Stockholm, Sweden; 5 Department of Oncology, Capio St Görans Sjukhus, Stockholm, Sweden; University of South Alabama Mitchell Cancer Institute, UNITED STATES

## Abstract

**Background:**

Knowledge about the relationship between PSA, age and ISUP grade group (ISUP) 1 prostate cancer can improve clinical and biological understanding of prostate cancer. We aimed to investigate the associations between PSA and age and the risk of ISUP 1 and ISUP ≥ 2 prostate cancer, respectively.

**Methods:**

We included 6 083 men aged 50–69 biopsied with a total of 72 996 individual biopsy cores from the prospective and population based Stockholm3 diagnostic study. We computed the risk of ISUP 1 and ISUP ≥ 2 prostate cancer and their respective associations with PSA and age. Since lower Gleason grades often are masked by higher grades in the overall Gleason score, we compared associations both for overall Gleason score and for Gleason on individual biopsy cores.

**Results:**

ISUP 1 prostate cancer was not significantly associated with PSA at diagnosis: odds ratios ranged from 0.82 (95%CI: 0.68–1.00) for PSA 3–4 ng/mL, 0.96 (95%CI: 0.79–1.16) for PSA 4–6 ng/mL, 0.95 (95%CI: 0.75–1.21) for PSA 6–10 ng/mL, and 0.92 (95%CI: 0.58–1.45) for PSA 10–15 ng/mL compared with PSA 2–3 ng/mL. Age was not significantly associated with risk of ISUP 1 cancer. This contrasts to the strong relationship between ISUP ≥ 2 prostate cancer and its respective associations with PSA and age.

**Conclusions:**

We find no significant association between the risk of ISUP 1 prostate cancer and PSA and age at diagnosis indicating that PSA contribution from ISUP 1 prostate cancer is closer to that of benign prostate tissue than to that of ISUP ≥ 2 prostate cancer.

## Introduction

Gleason grade 3+3 = 6 or International Society of Urological Pathology (ISUP) grade group (ISUP) 1 is the most common grade of prostate cancer, constituting approximately half of all diagnosed prostate cancers in countries with high uptake of prostate-specific antigen (PSA) testing.[1] The prostate specific mortality rate of men with a diagnosis of ISUP 1 prostate cancer is very low, comparable to that of men of the same age without a prostate cancer diagnosis.[2,3] Further, recent studies have shown that men with pure ISUP 1 cancer based on prostatectomy specimens have very low prostate cancer-specific mortality rates. In these studies, ISUP 1 cancers do not seem to be associated with a metastatic phenotype.[4] Even so, ISUP 1 cancer meets the histological definition of cancer and is associated with considerable psychological stress to the patient.[5,6]

The 2005 International Society of Urologic Pathology (ISUP) modified Gleason grading system (reporting global ISUP grade) is widely used.[7–9] By that definition Gleason pattern 3 has well-developed circumscribed glands and luminal differentiation with retained polarity of PSA-producing tumor cells. In Gleason pattern 4 there is only a partial glandular differentiation with some loss of cellular polarity. The tumor cells fail to form complete glands and aggregate in poorly formed, disrupted glands, merged glands and cribriform patterns that are less distinctly demarcated from the stroma.[10,11] This suggests that cells with a Gleason pattern 4 leak PSA through the disrupted cell membrane while cells with Gleason pattern 3 do not, or at least not to the same extent. Thus the association between PSA and ISUP 1 prostate cancer is different from the association between PSA and prostate cancer of ISUP 2 or higher.[12] It is well known that PSA is positively associated with Gleason pattern 4 and higher (ISUP ≥ 2 prostate cancer) and long-term risk of prostate cancer mortality.[13] In contrast, the risk of ISUP 1 biopsy findings has in fact been reported to decrease with increasing PSA.[14] However, because the global ISUP grade aggregates across grades on individual biopsy cores, biopsies with low-grade patterns might be masked by biopsies with higher-grade cancers, thus underestimating the true rate of low-grade cancer. In the present report, we therefore analyse both the overall ISUP grade and ISUP grades reported on each individual biopsy core (10 to 12 reported ISUP grades per man). This permits estimating ISUP grade specific disease rates, where higher ISUP grades do not mask lower grades and the relationship between PSA, age and ISUP grade can be more accurately estimated.

The aim of this paper was to study the relationship between PSA levels, age and the ISUP grade specific risk of prostate cancer in biopsy, both at the overall ISUP grade level as well as per individual biopsy core. This relationship is important in order to understanding prostate cancer aetiology and, ultimately, to whether ISUP 1 tumor cells should be labelled as cancer.[15,16]

## Materials and methods

### Participants

The Stockholm3 study (ISRCTN84445406) was a prospective and population-based study to compare the operating characteristics of the Stockholm3 prostate cancer risk prediction model to PSA for diagnostics of prostate cancer.[17,18] The study consisted of 59 149 men aged 50–69 years with no prior prostate cancer diagnosis. Men with a PSA concentration of at least 3 ng/mL or a Stockholm3 score of 10% or higher were referred to a urologist for biopsy. The biopsy procedure followed a standardized protocol using a 10 to 12 core systematic biopsy procedure (12 cores if the prostate volume was larger than 35 cc).^3^ All biopsy cores were graded individually by a single pathologist (L.E.). The pathologist was blinded to the patient’s PSA level, age, and other patient characteristics. We included all biopsied men in Stockholm3 with a PSA between 2 and 15 ng/mL, in total 6 083 men.

### Statistical methods

We estimated the association between PSA level, age, and biopsy outcome using two different models:

1. **Overall ISUP grade model:** Using multinomial logistic regression, we modelled the probability of different biopsy outcomes (benign finding, ISUP 1, ISUP ≥ 2) for a man when undergoing systematic biopsy, by PSA level and age. We adjusted for known risk factors for prostate cancer in our model: digital rectal exam, previous negative biopsy and family history of prostate cancer. The equation for the multinomial logistic regression model is:
  $${outcome}_{biopsy}=\beta_{0}+\beta_{1}\;\log\left(PSA\right)+\beta_{2}age+\beta_{3}DRE+\beta_{4}prevBiopsy+\beta_{5}prostateVolume+\beta_{6}familyHistory$$
2. **Biopsy core ISUP grade model:** We estimated the proportion of individual biopsy cores classified as benign, ISUP 1, and ISUP ≥ 2 within a man, also using logistic regression. Each man represents a single entry in the analysis, with age and PSA as independent variables and the dependent variable as the proportions of benign cores, ISUP 1 cores, and ISUP ≥ 2 cores within the biopsied man, respectively. We again adjusted for known risk factors for prostate cancer in the model: digital rectal exam, previous negative biopsy and family history of prostate cancer. In the analysis for the risk of ISUP 1 cancer we also adjusted for ISUP ≥ 2 cancer in any of the other biopsy cores within a man to account for the cases with a ISUP 1 core where another biopsy core includes a ISUP ≥ 2 cancer. The equation for the multinomial logistic regression model is similar to the previous model, with a different outcome variable Y, where Y represents the proportion of different biopsy outcomes within each man:

$$Y=\beta_{0}+\beta_{1}PSA\_category+\beta_{2}age+\beta_{3}DRE+\beta_{4}prevBiopsy+\beta_{5}prostateVolume+\beta_{6}familyHistory$$

To ease interpretation and to allow for nonlinear effects, we discretized PSA into the following categories: 2–3 ng/mL, 3–4 ng/mL, 4–6 ng/mL, 6–10 ng/mL, and 10–15 ng/mL. From both models we calculated the odds ratios for the risk of ISUP grade specific biopsy outcomes by PSA levels and age and the respective 95% confidence intervals. In addition, we used the models to estimate the risk of Gleason specific outcomes from both models for PSA levels 2–15 ng/mL at age 50, 60, and 70 years.

The predictor variables in our model, PSA and age are known to be associated with prostate cancer, high PSA is a considerably good indicator for high-grade prostate cancer and older men are at higher risk of being diagnosed with prostate cancer. The covariates in our models are also known risk factors for prostate cancer and possible confounders that we feel that need to be adjusted for. Abnormal digital rectal exam (DRE) would indicate that a cancer has started to grow outside the prostate, men with a previous negative biopsy are at lower risk of a positive biopsy, smaller prostate volume has been shown to increase the risk of cancer in biopsy and a positive family history of prostate cancer has also been linked with higher risk of prostate cancer.

We used these two models to explore the difference in a final outcome of biopsy (highest ISUP grade) and the frequency of different biopsy outcomes within each man (Y variable). By doing so, we can explore if a lower grade ISUP prostate cancer that is hidden under the outcome of higher grade prostate cancer is associated with PSA levels and age.

We used R statistical software v.3.5.1 (R Foundation for Statistical Computing, Vienna, Austria) for all analyses.

### Consent and approval

The Stockholm3 study was approved by the research ethics board at the Regional Ethics Testing Board, Stockholm; EPN DnR 2012/438-31/3. The registration number for the study is NCT03639649/ ISRCTN84445406. All study participants have given written informed consent (as outlined in the PLOS consent form) to publish these case details.

## Results

A total of 72 996 biopsy cores in 6 083 men were included in our study population (Tables 1 and 2).

**Table 1:** Study population characteristics.

Characteristics per man						
	n	ISUP grade 1 (%)	ISUP grade 2 (%)	ISUP grade 3 (%)	ISUP grade 4 or higher (%)	Benign biopsy (%)
**Number of men**	6083	1331 (22)	629 (10)	193 (3)	145 (3)	3785 (62)
PSA* **at inclusion**						
2 to 3	1007	245 (24)	99 (10)	28 (3)	13 (1)	622 (62)
3 to 4	1960	411 (21)	166 (9)	48 (2)	39 (2)	1296 (66)
4 to 6	1827	403 (22)	199 (11)	56 (3)	42 (2)	1127 (62)
6 to 10	787	151 (19)	110 (14)	41 (5)	32 (4)	453 (58)
10 to 15	182	28 (15)	32 (18)	15 (8)	18 (10)	89 (49)
**Age (years)**						
50–54	536	119 (22)	60 (11)	10 (2)	11 (2)	336 (63)
55–59	1008	240 (24)	85 (8)	29 (3)	12 (1)	642 (64)
60–64	1688	356 (21)	175 (11)	54 (3)	37 (2)	1066 (63)
65–70	2851	616 (21)	309 (11)	100 (4)	85 (3)	1741 (61)
**First degree relative with prostate cancer**						
Yes	901	242 (26)	122 (14)	35 (4)	25 (3)	477 (53)
No	5182	1089 (21)	507 (10)	158 (3)	120 (2)	3308 (64)
**Men with a previous negative biopsy**						
Yes	400	52 (13)	16 (4)	5 (1)	3 (1)	324 (81)
No	5683	1279 (23)	613 (11)	188 (3)	142 (2)	3461 (61)
**Prostate Volume (mL)**						
<35	1996	501 (25)	300 (15)	96 (5)	61 (3)	1038 (52)
35–50	2224	484 (22)	211 (9)	67 (3)	59 (3)	1403 (63)
>50	1863	346 (19)	118 (6)	30 (2)	25 (1)	1344 (72)
**Digital rectal exam**						
Abnormal	526	106 (20)	104 (20)	53 (10)	39 (7)	224 (43)
Normal	5557	1225 (22)	525 (9)	140 (3)	106 (2)	3561 (64)

Biopsied men in the Stockholm3 study with PSA levels 2–15 ng/mL

* Prostate Specific-Antigen

**Table 2:** Biopsy outcome per each biopsy core.

Characteristics per biopsy core						
	n	ISUP grade 1 (%)	ISUP grade 2 (%)	ISUP grade 3 (%)	ISUP grade 4 or higher (%)	Benign biopsy (%)
**Number of biopsies**	72996	4350 (6)	1518 (2)	559 (1)	599 (1)	65970 (90)
PSA* **at inclusion**						
2 to 3	12084	703 (6)	221 (2)	67 (0.5)	63 (0.5)	11030 (91)
3 to 4	23520	1232 (5)	361 (2)	117 (1)	130 (1)	21680 (92)
4 to 6	21924	1329 (6)	440 (2)	150 (1)	166 (1)	19839 (90)
6 to 10	9444	662 (7)	339 (4)	141 (1)	147 (2)	8155 (86)
10 to 15	2184	149 (7)	121 (6)	74 (3)	91 (4)	1749 (80)
**Age (years)**						
50–54	6432	464 (7)	153 (2)	28 (1)	45 (1)	5742 (89)
55–59	12096	748 (6)	213 (2)	83 (1)	64 (1)	10988 (90)
60–64	20256	1136 (6)	408 (2)	171 (1)	149 (1)	18392 (90)
65–70	34212	2002 (6)	744 (2)	277 (1)	341 (1)	30848 (90)
**First degree relative with prostate cancer**						
Yes	10812	854 (8)	313 (3)	106 (1)	117 (1)	9422 (87)
No	62184	3496 (6)	1205 (2)	453 (1)	482 (1)	56548 (90)
**Men with a previous negative biopsy**						
Yes	4800	131 (3)	31 (1)	15 (0.5)	12 (0.5)	4611 (95)
No	68196	4219 (6)	1487 (2)	544 (1)	587 (1)	61359 (90)
**Prostate Volume (mL)**						
<35	23952	1909 (8)	758 (3)	285 (1)	272 (1)	20728 (87)
35–50	26688	1547 (6)	519 (2)	197 (1)	226 (1)	24199 (90)
>50	22356	894 (4)	241 (1)	77 (0.5)	101 (0.5)	21043 (94)
**Digital rectal exam**						
Abnormal	6312	570 (9)	337 (5)	182 (3)	169 (3)	5054 (80)
Normal	66684	3780 (6)	1181 (2)	377 (1)	430 (1)	60916 (90)

Biopsy cores from all biopsied men in the study population with PSA levels 2–15 ng/mL.

* Prostate Specific-Antigen

Most men had a benign global biopsy result (62%), 22% were diagnosed with ISUP 1 cancer and 16% with ISUP ≥ 2 prostate cancer (Table 1). Men with high PSA levels (10 to 15 ng/mL) had the highest rate of ISUP ≥ 2 prostate cancer (36%) compared to men with low PSA levels (2 to 3 ng/mL) (14%). The rate of ISUP 1 cancers decreased with increasing PSA levels ranging from 24% for the lowest PSA category to 15% for the highest PSA category. The rate of ISUP 1 prostate cancer was similar for different age groups. However the rate of ISUP ≥ 2 prostate cancer increased slightly with increasing age, from 15% for the youngest men up to 18% for the oldest men.

Six percent of the individual biopsy cores were graded with ISUP 1 prostate cancer, 4% were ISUP ≥ 2 prostate cancers and 90% were benign (Table 2). The number of ISUP 1 prostate cancer were similar with increasing PSA level; for men with PSA 1 to 5 ng/mL, 6% of the cores were graded with ISUP 1 prostate cancer compared to 7% among men with PSA levels 6 to 15 ng/mL. In contrast, the increase was much larger for the ISUP ≥ 2 prostate cancer, where only 3% of the men with PSA level 2–3 ng/mL were diagnosed with ISUP ≥ 2 cancer compared to 13% among the men with PSA level 10–15 ng/mL.

### Overall ISUP grade

**Fig 1:**
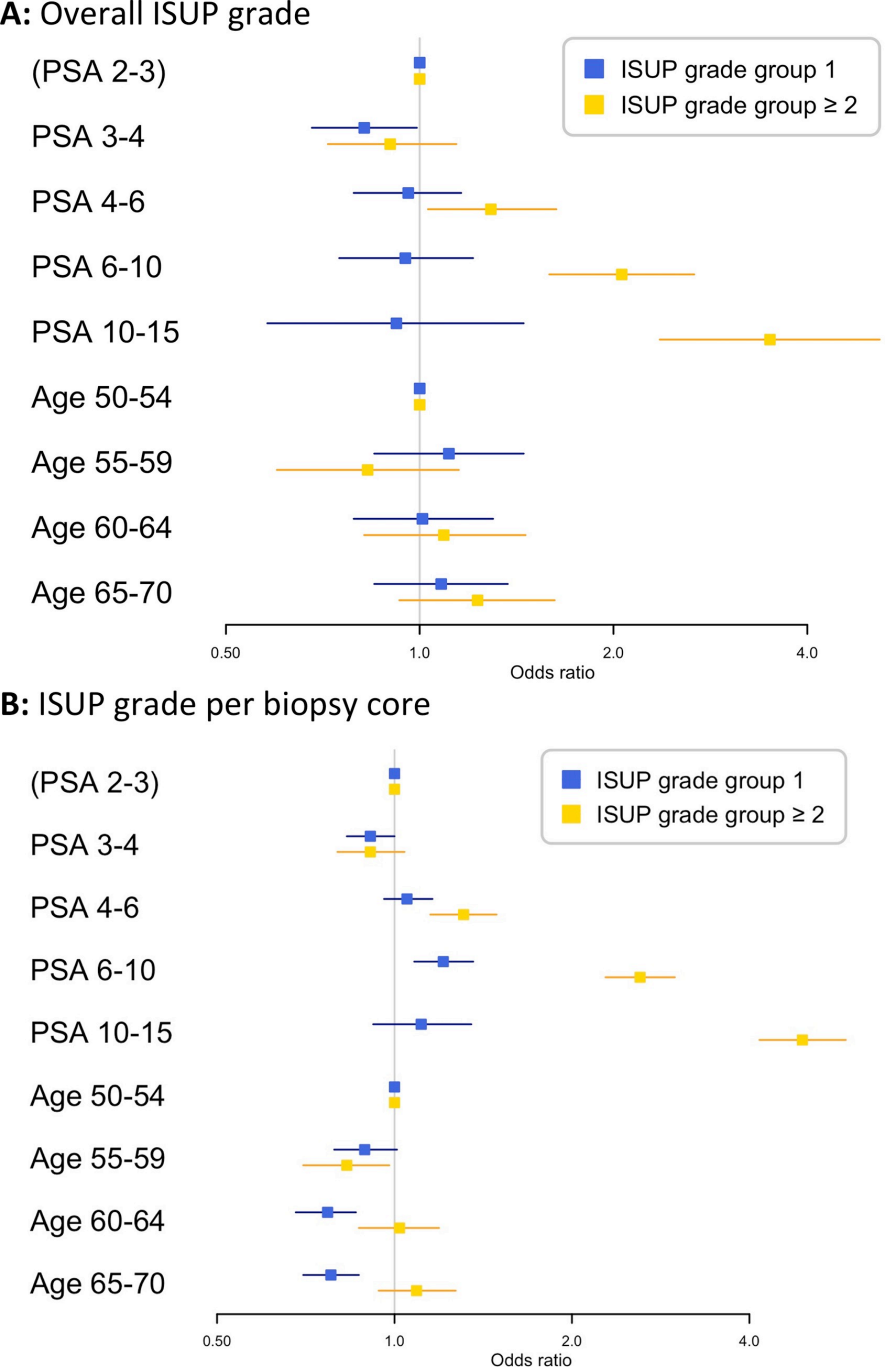
Forest plots showing the odds ratios with 95% confidence intervals for the risk of ISUP grade specific outcomes in biopsy. The reference level is a man with PSA of 2–3 ng/mL and age group 50–54 years and the plot shows the odds ratios comparing these groups to men in the reference level group. **A: Overall ISUP grade. B: ISUP grade per biopsy core** (all reported ISUP grade groups for 10 to 12 cores of all men).

PSA was not significantly associated with the risk of overall ISUP 1 prostate cancer, with odds ratios ranging from 0.82 (95%CI: 0.68–1.00) for a man with PSA level from 3 to 4 ng/mL, 0.96 (95%CI: 0.79–1.16) for a man with PSA level from 4 to 6 ng/mL, 0.95 (95%CI: 0.75–1.21) for PSA 6–10 ng/mL, and 0.92 (95%CI: 0.58–1.45) for PSA 10–15 ng/mL compared to a man with PSA level from 2 to 3 ng/mL (Fig 1A). However, increasing PSA level was significantly associated with higher risk of ISUP ≥ 2 prostate cancer with odds ratios ranging from 0.90 (95%CI: 0.72–1.14) for PSA levels 3 to 4 ng/mL, 1.29 (95%CI: 1.03–1.63) for PSA levels 4 to 6 ng/mL, 2.06 (95%CI: 1.59–2.67) for PSA levels 6 to 10 ng/mL and 3.50 (95%CI: 2.36–5.18) for PSA levels 10 to 15 ng/mL compared to men with a PSA from 2 to 3 ng/mL. We found similar patterns for the association of age with risk of ISUP 1 and ISUP ≥ 2 prostate cancer (Fig 1A), where increasing age had no significant effect on the risk of ISUP 1 cancer while higher age was significantly associated with higher risk of ISUP ≥ 2 cancer.

**Fig 2:**
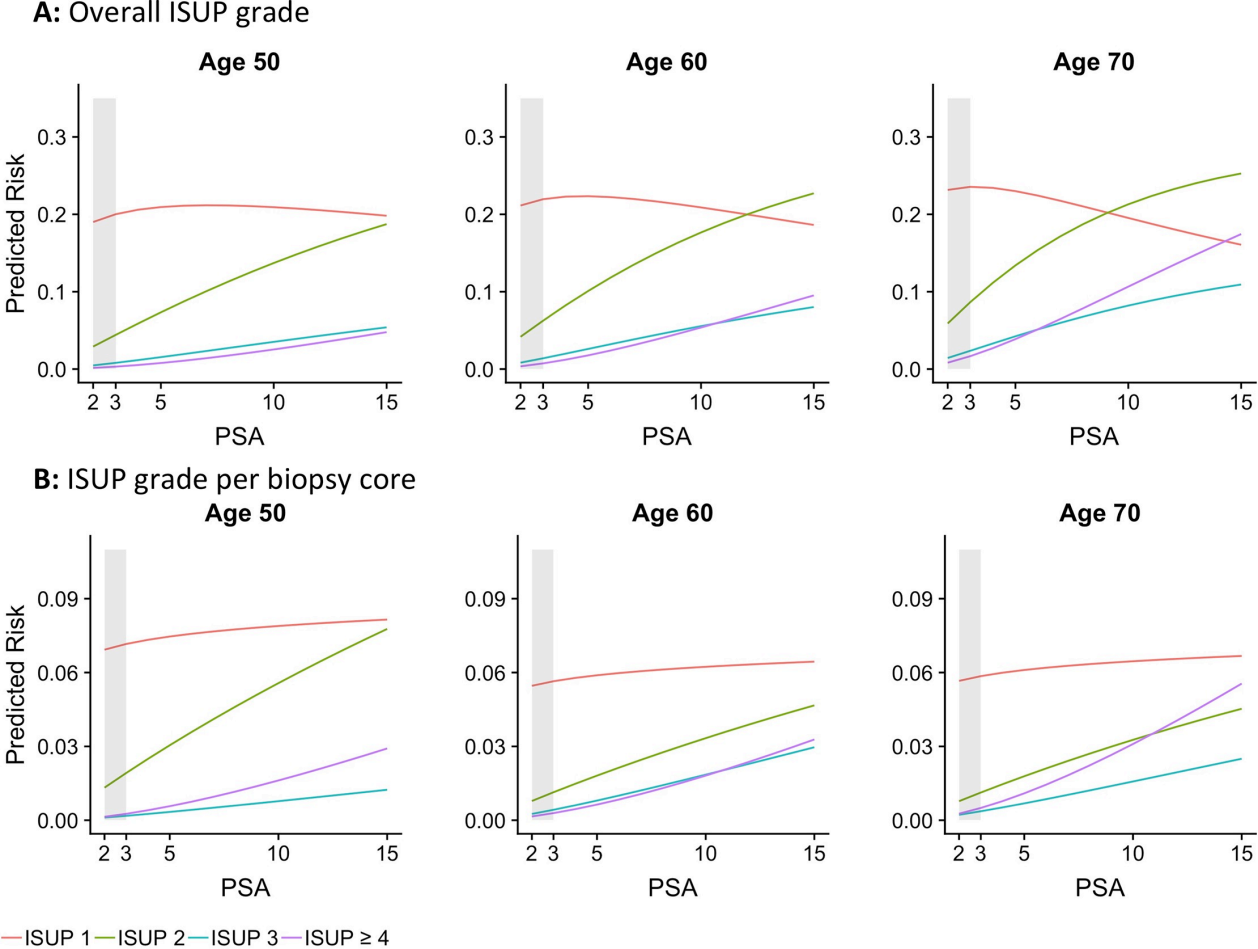
**A: Overall ISUP grade.** Overall ISUP grade reported, one grade per man. **B: Biopsy core level.** Risk of ISUP grade group per 10–12 biopsy cores per man.

Fig 2A shows the estimated risk of ISUP specific prostate cancer depending on PSA at age 50, 60 and 70 years. The risk of ISUP 1 cancer decreases for increasing PSA levels for ages 60 and 70 years, while it decreases slightly more for the oldest group with increasing PSA level, due to the fact that higher ISUP grade cancers mask the rate of ISUP 1 cancer on an aggregated level. The well-known increased risk of ISUP ≥ 2 cancer with increasing age and PSA is clear in Fig 2A, in particular for ISUP ≥ 3.

### Biopsy core level

When analysing individual biopsy cores to avoid higher grade cancer masking lower grade cancer, PSA was not significantly associated with the risk of ISUP 1 cancer (Fig 1B) for three of the four PSA groups, with odds ratios ranging from 0.91 (95%CI: 0.83–1.00) for PSA level 3 to 4 ng/mL, 1.05 (95%CI: 0.96–1.16) for PSA level 4 to 6 ng/mL, 1.21 (95%CI: 1.08–1.36) for PSA level 6 to 10 ng/mL and 1.11 (95%CI: 0.92–1.35) for PSA level 10 to 15 ng/mL compared to men with PSA from 2 to 3 ng/mL. For the risk of ISUP ≥ 2 prostate cancer, this trend was markedly stronger with odds ratios ranging from 0.91 (95%CI: 0.80–1.04) for PSA levels 3 to 4 ng/mL, 1.31 (95%CI: 1.15–1.49) for PSA levels 4 to 6 ng/mL, 2.61 (95%CI: 2.28–2.99) for PSA levels 6 to 10 ng/mL and 4.92 (95%CI: 4.16–5.83) for PSA levels 10 to 15 ng/mL compared to men with PSA from 2 to 3 ng/mL. With increased age the risk of ISUP 1 prostate cancer decreased slightly (ORs ranging from 0.89 (95%CI: 0.79–1.01) to 0.78 (95%CI: 0.70–0.87)), while higher age was not significantly associated with increased risk of ISUP ≥ 2 cancer on an individual biopsy core level.

Fig 2B shows the estimated risk of ISUP grade specific outcomes per biopsy core depending on PSA level at age 50, 60 and 70 years. There was a slight increasing trend in the risk of ISUP 1 cancer with increasing PSA levels whereas the risk is slightly decreasing with age. The risk of higher ISUP grade cancer increases with increasing PSA levels for all ages, however risk of ISUP ≥ 4 cancer increases more rapidly for men aged 60 and 70 years.

## Discussion

We used global ISUP grade group (overall ISUP grade at diagnosis) as well as ISUP grade group assigned to individual prostate biopsy cores to study the association between PSA, age and ISUP grade specific prostate cancer.

### Overall ISUP grade

We found that there is about a 20% risk that a man is diagnosed with ISUP 1 prostate cancer when undergoing a 10- or 12-core systematic biopsy, regardless of the man’s PSA level and age. This contrasts to the strong relationship between PSA and ISUP ≥ 2 cancer, supporting previous observations that that ISUP ≥ 2 cancer is more likely to leak PSA into the blood than ISUP 1 cancer.[12] Most of these ISUP 1 cancers are likely to be clinically insignificant and illustrate the problem of over-diagnosis. This highlights a well-known problem with prostate cancer diagnostics and underlines the importance of avoiding unnecessary biopsies. As risk stratification tools[18–23] and magnetic resonance imaging (MRI) together with targeted biopsies are increasingly being introduced,[24–26] this high proportion of ISUP 1 cancer will likely decrease.

### Biopsy core level

We did not observe a significant association between increasing PSA and higher proportion of biopsy cores with ISUP 1 cancer for three out of four PSA groups. These results strengthen our theory that PSA contribution of ISUP 1 prostate cancer is closer to benign tissue than ISUP ≥ 2 cancer. This contrasts to the strong relationship between increasing PSA and the risk of ISUP ≥ 2 cancer. The weak nonsignificant association between PSA and proportion of biopsy cores with ISUP 1 cancer may potentially be that cancers that are misdiagnosed as ISUP 1 in biopsy but in truth have a higher grade due to the systematic biopsy cores missing the area of the prostate containing the highest grade cancer. In fact, data on 944 men in the Stockholm3 study that had a prostatectomy after a prostate cancer diagnosis, 164 (17.3%) men had an ISUP upgrade after operation. An analysis on these men shows that upgrading after surgery is associated with higher average PSA level then among other biopsied men within the Stockholm3 study.

ISUP 1 prostate cancer is found at an equal probability irrespective of a man’s age (Figs 1B and 2B). This observation begs the question: When does pattern 3 start to develop in a man’s prostate and is it possibly there from very early ages? Based on autopsy material, studies from several authors have been published on the incidence of ISUP 1 and ISUP ≥ 2 cancer in different age groups.[27–29] These studies show that prostate cancer is found in men from the early age of 20 years and upwards and that ISUP 1 prostate cancer does not have the same strong association with increasing age as ISUP ≥ 2 cancer.

The results for ISUP 1 cancer are in contrast to the strong association between PSA, age and risk of ISUP ≥ 2 prostate cancer, where the risk of higher Gleason grade prostate cancer increases with increased levels of PSA in the blood and higher age.

### Strengths and limitations

Strengths of this study include the well-controlled, prospective and population based design covering of a large random subsample of screening aged men 50–69 years of age in Stockholm, the uniquely consistent pathology assessment (all biopsy cores were graded by a single pathologist), and the fact that the pathologist was blinded to patient characteristics (including PSA and age). In the study, all men were biopsied using 10 to 12 core systematic biopsy. For the purpose of estimating the presence of ISUP 1 cancer in the prostate, systematic biopsies likely give a more representative view of the prostate cells rather than sampling only from the area most likely to contain higher grade cancer, as would be done in MRI-targeted biopsies.

The Stockholm3 study has some limitations. Since the study focused on men in the screening age 50–69 years old, we do not know the rate of ISUP 1 prostate cancer in younger nor older men. The study was performed in Stockholm, Sweden where most participants are of northern European descent, who have a high risk of prostate cancer. However, evidence suggests that prostate cancer outcomes are similar in Caucasian populations in other parts of the world.

## Conclusions

The ISUP 1 cancer risk ascertained at diagnosis and on a biopsy core level is not associated with age and PSA, supporting the view that the PSA contribution from ISUP 1 prostate cancer is closer to that of benign prostate tissue than to that of ISUP ≥ 2 prostate cancer.
